# Pleuritis due to *Mycobacterium xenopi* without pulmonary infection

**DOI:** 10.1099/acmi.0.000328

**Published:** 2022-03-22

**Authors:** Keren Bachar, Tiberiu Shulimzon, Efrat Ofek, Michael J. Segel

**Affiliations:** ^1^​ Institute of Pulmonology, Sheba Tel-HaShomer Medical Center, Ramat Gan, Israel; ^2^​ Department of Pathology, Sheba Tel-HaShomer Medical Center, Ramat Gan, Israel; ^3^​ Sackler Faculty of Medicine, Tel-Aviv University, Tel-Aviv, Israel

**Keywords:** *Mycobacterium xenopi*, pleuritis, pulmonary infection

## Abstract

Nontuberculous mycobacteria (NTM) may cause pulmonary and extra-pulmonary disease in both immunocompetent and immunocompromised patients. Pleuritis is an uncommon manifestation on NTM disease, and pleuritis caused by *Mycobacterium xenopi* has only been described once before. Because it is considered to be an environmental contaminant, isolation of *M. xenopi* from bronchopulmonary secretions or other sites is often dismissed. The disease caused by *M. xenopi* is usually a pulmonary infection and typically occurs in severely immunocompromised individuals or in immunocompetent patients with an underlying chronic lung disease. We describe an unusual case of pleuritis caused by *M. xenopi* in a patient without an underlying chronic lung disease and with no evidence of a concurrent *M. xenopi* pulmonary infection.

## Introduction

Nontuberculous mycobacteria (NTM) are a group of organisms that are ubiquitous in the environment and may cause pulmonary and extra-pulmonary disease in both immunocompetent and immunocompromised patients [1, 2]. While pulmonary disease accounts for the vast majority of NTM diseases, pleuritis caused by these organisms is uncommon, making its diagnosis and treatment challenging [3–5].

The slow-growing *Mycobacterium xenopi* is an obligate thermophilic organism that has been isolated from environmental water, soil, tap-water systems, showerheads and hot-water tanks [6–8]. It is the second most common cause of NTM lung disease in Canada, the UK, and some parts of Europe and The Middle East [9–11]. Disease caused by *M. xenopi* is usually a pulmonary infection mimicking tuberculosis and typically occurs in patients with an underlying lung disease or in immunocompromised patients, especially HIV patients [12–14]. Extra-pulmonary manifestations and disseminated disease are rare but have been reported [15–17].

Pleuritis caused by *M. xenopi* has only been described once before in a patient with a concurrent pulmonary infection [18]. We report a case of pleuritis caused by *M. xenopi* in a patient without underlying chronic lung disease and with no evidence of a concurrent *M. xenopi* pulmonary infection.

## Case report

A 69-year-old male ex-smoker (five packs/year) presented to our clinic with a routine spirometry showing a mild restriction (FEV1=2.11 l, 72 % of predicted; FVC=2.69 l, 69 % of predicted; FEV1/FVC=0.78). He had no respiratory complaints, including cough, sputum production or dyspnoea, and denied any other symptoms, such as malaise, fever or weight loss. His medical history included hypertension, diabetes and dyslipidaemia, for which he received medications, and past surgeries for nephrolithiasis, ocular melanoma and basal cell carcinoma of the scalp. Ten years prior to presentation he had a positive tuberculin skin test (17 mm), for which he was not treated.

**Fig. 1.**
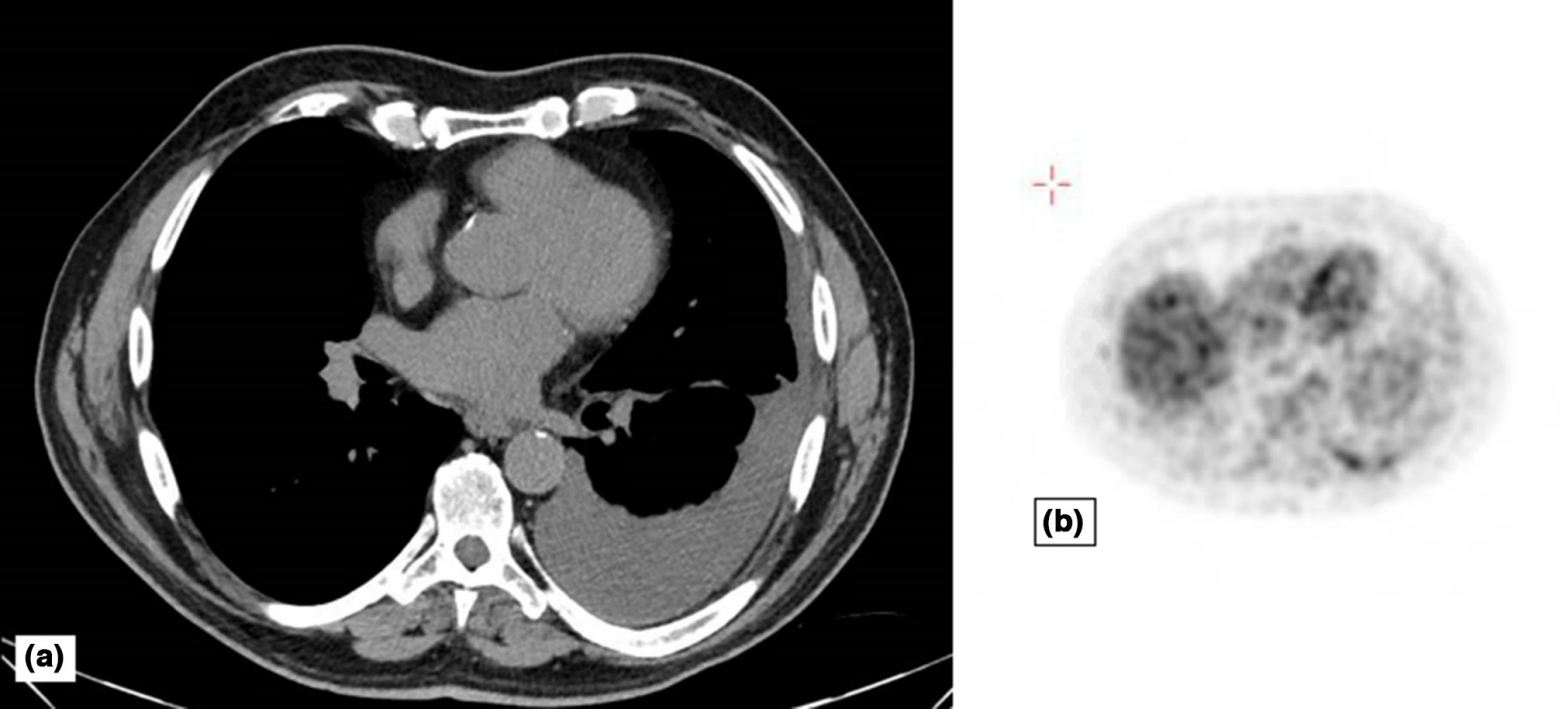
FDG-PET/CT obtained on patient presentation. (a) Left medium-sized pleural effusion. (b) FDG uptake in the left basal dorsal pleura.

Physical examination revealed diminished breath sounds and dullness to percussion in the left lung base, and the chest roentgenogram showed a medium-sized left pleural effusion. Laboratory tests showed a C-reactive protein (CRP) level of 53 mg dl^−1^ (reference range, 0 to 5). Complete blood count, including leukocyte and differential count, was normal (9460 cell μl^−1^ total leukocytes with 59 % neutrophils, 30 % lymphocytes, 9 % monocytes,and 1 % each of eosinophils and basophils) and so were his renal and liver functions. An FDG-PET/CT was performed, which showed a left pleural effusion with FDG-avid thickening of the basal dorsal pleura (shown in Fig. 1). There neither lymphadenopathy nor other abnormalities in the lungs or in the rest of the body.

Thoracocentesis was performed and a total of 700 cc of clear fluid was drained. Analysis revealed an exudate with lymphocyte predominance (93 %). Lactic dehydrogenase (LDH) and protein levels were mildly elevated. Adenosine deaminase level was not measured. Cytological examination did not show malignant cells. No bacteria were detected in bacterial cultures or by Ziehl–Neelsen staining of the fluid and polymerase chain reaction (PCR) for TB was negative. Serum immunoglobulin and anti-interferon-gamma auto-antibody levels were not performed. However, *M. abscessus* was isolated in one out of three sputum cultures.

**Fig. 2.**
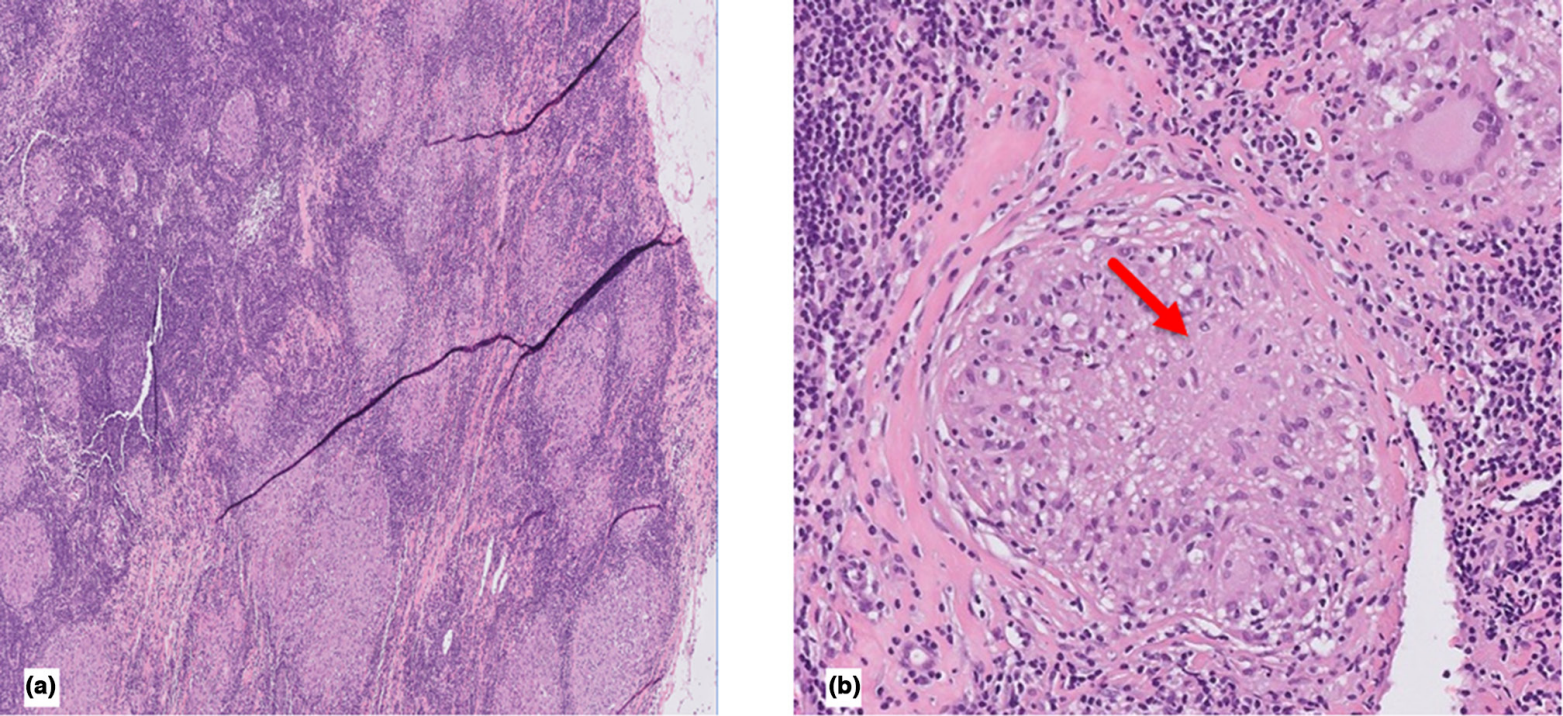
HE stain of the pleural biopsies obtained from thoracoscopy. (a) Well-formed epithelioid and giant cell granulomas (×5). (b) Tiny foci of central necrosis (arrow) (×20).

Thoracoscopic inspection of the left pleural cavity revealed adhesions at the lung base, in addition to multiple small white lesions of the parietal pleura in this area. Pleural biopsies were taken, which demonstrated well-formed epithelioid and giant cell granulomas with tiny foci of central necrosis in some of them, consistent with granulomatous pleuritis (shown in Fig. 2). Cultures that were taken from the pleura and from the effusion during the surgery grew *M. xenopi *. Consequently, the *M. abscessus* in the sputum was considered a contamination, and the patient was diagnosed with granulomatous pleuritis due to *M. xenopi *.

Antimycobacterial therapy was initiated with isoniazid, rifampin and ethambutol. However, treatment was stopped after 2 months due to arthralgias. By that time, the serum CRP level was 2 mg dl^−1^ and a repeat chest CT showed resorption of the pleural effusion with some residual pleural thickening. At the last follow-up 9 months after the initial presentation, the patient feels well and a chest radiograph shows mild pleural thickening.

## Discussion

Unlike pleural involvement in *Mycobacterium tuberculosis* infection, NTM pleurisy is not common, and is reported to account for <6 % of NTM infections [1–4]. The most common isolate in the reported cases of NTM pleurisy is *Mycobacterium avium* complex (MAC), followed by *Mycobacterium kansasii* and *Mycobacterium intracellulare* [3, 5, 18, 19]. Isolation of *M. xenopi* from the pleural space has previously only been described in one patient, reported in a case series from Taiwan [18]. The pathogenesis of NTM pleuritis, although unclear, is considered to be similar to that of tuberculous pleuritis, i.e. the perforation of a subpleural focus or the direct spread of inflammation to the pleura from a concurrent pulmonary NTM disease [3, 19].

The clinical significance of laboratory isolation of *M. xenopi* may be uncertain. Since it is present in the environment, isolation of *M. xenopi* from bronchopulmonary secretions or other sites is often dismissed, and it is considered an environmental contaminant [14]. Although it is generally assumed that when an NTM is isolated from a closed space, such as the pleural cavity, it is responsible for the observed pathological changes, NTM may gain entry to a pleural cavity containing an effusion of an unrelated aetiology, such as congestive heart failure or metastatic cancer [20]. In the present case, the patient did not have such an underlying disease, and the granulomas seen in his pathological specimens were typical of an NTM infection, making *M. xenopi* pleuritis the most likely diagnosis.

*M. xenopi* infections usually arise in severely immunocompromised individuals or in immunocompetent patients with underlying chronic lung disease [13, 14]. In most cases, the disease caused by *M. xenopi* is a pulmonary (parenchymal) infection with a clinical presentation that closely mimics tuberculosis, including productive cough, weight loss, fever, night sweats and weakness. Typical radiological findings also mimic tuberculosis with upper lobe cavitations and parenchymal infiltrates [1, 12]. In immunocompromised individuals *M. xenopi* may also occur as an opportunistic infection and spread haematogenously, causing disseminated disease and infection of normally sterile sites, such as arthritis and osteomyelitis [17, 21–24]. Another group of patients in whom *M. xenopi* infection has been described are patients after invasive procedures or hospitalizations, suggesting iatrogenic and nosocomial origins due to the colonization of the organism in hospital water systems [6–8, 15, 25, 26]. Surprisingly, our patient did not have an underlying lung disease, he was not significantly immunosuppressed (only mild and controlled diabetes) and he had not undergone any invasive procedure in the chest or experienced a recent hospitalization. Moreover, he did not have any radiological findings of a pulmonary NTM disease, and *M. xenopi* was not isolated from his sputum. The only other reported case in the literature of *M. xenopi* pleuritis had concurrent pulmonary infection [18], and we found only a few cases of NTM pleuritis of any species with no obvious pulmonary involvement [27–33].

In conclusion, *M. xenopi* is a very rare cause of pleuritis, with the presented case being the second reported in the literature. Another unusual feature of this case is the absence of pulmonary parenchymal involvement, previously reported in very few cases of NTM pleuritis. Despite the rarity of this presentation, and the frequency of its isolation as a laboratory contaminant or a colonizing agent, the pathogenic potential of *M. xenopi* should not be disregarded.
